# Serum Bilirubin Is Correlated With the Progression of IgA Vasculitis With Nephritis

**DOI:** 10.3389/fmed.2021.596151

**Published:** 2021-06-08

**Authors:** Jiaxing Tan, Gaiqin Pei, Yicong Xu, Tengyue Hu, Li Tan, Zhengxia Zhong, Padamata Tarun, Yi Tang, Wei Qin

**Affiliations:** ^1^Division of Nephrology, Department of Medicine, West China Hospital, Sichuan University, Chengdu, China; ^2^West China School of Medicine, Sichuan University, Chengdu, China

**Keywords:** IgA vasculitis with nephritis, bilirubin, cohort study, prognosis, biomarker

## Abstract

**Background:** Bilirubin has been identified as an endogenous antioxidant and cellular protectant. The present study was performed to clarify the potential influence of serum bilirubin on IgA vasculitis with nephritis (IgAV-N).

**Methods:** One hundred and eighty-nine IgAV-N patients over 14 years old were enrolled. The patients were divided into two groups by the optimum cut-off value calculated by ROC curve. The composite endpoints were defined as a 60% decline in estimate glomerular filtration rate (e-GFR), end-stage renal disease (ESRD) and/or death. Kaplan-Meier (K-M) analysis and multivariate Cox analysis were carried out to determine the predictors for renal outcomes. In order to eliminate the influence of different baseline data, a 1:2 propensity score (PS) match was performed to make the results comparable and convictive.

**Results:** The baseline data suggested that patients in low serum bilirubin group had significantly higher levels of systolic blood pressure, proteinuria, serum creatinine and crescent formation ratio and lower levels of serum albumin and hemoglobin. Renal survival analysis indicated that lower serum bilirubin levels were significantly correlated with a poorer prognosis. Multivariate Cox analysis demonstrated that the higher level of serum bilirubin was an independent protective factor for renal survival (HR, 0.172; 95% CI, 0.030–0.991; *P* = 0.049). After PS matching, the baseline characters of two groups had no statistical differences. Similar outcomes were demonstrated in K-M curve and the multivariate Cox analysis.

**Conclusion:** Elevated bilirubin levels might be related to the favorable renal outcomes.

## Introduction

IgA vasculitis with nephritis (IgAV-N) is one of the most severe complications of IgA vasculitis (IgAV) and the long-term prognosis of IgAV are mostly determined by the severity of renal lesions, owing that IgAV-N can cause end-stage renal disease (ESRD) and even death (1). The pathophysiological processes of IgAV-N have not been fully elucidated yet, however, it is widely acknowledged that the deposits of immune complexes (IC) in glomeruli might contribute to the progression of IgAV-N. Deposited IC may further cause oxidant injury, inflammation reaction and endothelial damage, all of which play a pivotal part in the renal impairment (2). The pathogenesis is much similar to primary IgA nephropathy (IgAN), therefore IgAV-N is also considered as secondary IgAN (1).

Bilirubin has been identified as an endogenous antioxidant and cellular protectant, with potent abilities of complement inhibition and anti-inflammation (3). Previous literatures have demonstrated that low serum bilirubin concentration is related to poor outcomes of diabetic nephropathy, IgAN and other chronic kidney diseases (CKD) (4–6). Additionally, animal experiments have reported that bilirubin might protect against diabetic nephropathy (7). Given the similarities in IgAV-N and IgAN, serum bilirubin may have a likely impact on IgAV-N, but no study has proved it. Therefore, the present study was conducted to evaluate whether serum bilirubin levels could serve as an independent predictor of IgAV-N prognosis and to clarify the potential influence of serum bilirubin on the progression of IgAV-N.

## Methods

This study was approved by the Ethics Committee of the West China Hospital of Sichuan University and retrospectively registered in Thai Clinical Trials Registry. The registration number is TCTR20180313004.

### Subjects

One hundred and eighty-nine adolescent and adult patients with IgAV-N proven by renal biopsy in West China Hospital of Sichuan University between October 2010 and June 2017 were enrolled prospectively in this study. Diagnostic criteria of IgAV was based on guidelines from the American College of Rheumatology (ACR) database and methodology (8). Renal biopsy was performed in patients who had hematuria, proteinuria and/or renal dysfunction. Notably, all subjects enrolled in our cohort were over 14 years old. Individuals with systemic diseases including systemic lupus erythematosus, autoimmune hemolytic anemia, malignant neoplasm, acute/chronic liver disease, congenital kidney disease, and inherited kidney disease were excluded. Written informed consents were obtained.

### Grouping and Matching

Total bilirubin levels were detected at baseline and IgAV-N patients were divided into high serum bilirubin level group (HsB) and low serum bilirubin level (LsB) group based on the optima cutoff value calculated by receiver operating characteristic (ROC) curve, which was frequently used in the medical statistics (9). In order to eliminate the influence of differences at baseline on the renal outcome, we performed a 1:2 propensity score (PS) match based on the greedy matching algorithm (10, 11). Covariates including age, gender, systolic blood pressure (SBP), diastolic blood pressure (DBP), proteinuria, urinary red blood cell (u-RBC), albumin, serum creatinine, hemoglobin and pathological findings, were considered in this multi-logit regression model, in order to make the results comparable. The caliper used for PS matching was within 0.2 SD of logit PS.

### Measurement

All patients enrolled in this study were followed up regularly for at least 6 months or shorter if they had reached the endpoints of observation in our medical center. Their clinical histories and out-patient records were assessed. Venous blood samples were taken from IgAV-N patients fasting overnight in the early morning at each time of follow up for measurement of serum total bilirubin, albumin, creatinine, uric acid, triglyceride and cholesterol. Serum total bilirubin was measured by vanadate oxidizing method and the normal reference range was 5.0–28.0 umol/L. Estimate glomerular filtration rate (e-GFR) was calculated using chronic kidney disease epidemiology collaboration (CKD-EPI) formula. Besides, proteinuria levels and hematuria levels (u-RBC) were also recorded. Anemia was diagnosed by a hemoglobin of <120 g/L in men while in women, it was <110 g/L. Considering that there was no universally accepted pathological classification of adult-onset HPSN, the pathology results in this study were largely based on Oxford classification of IgAN. Renal pathological lesions were classified as mesangial proliferation (M), endocapillary proliferation (E), segmental glomerulosclerosis (S), tubular atrophy/interstitial fibrosis (T) and fibrocellular/cellular crescents (C) (12).

### Treatment and Definitions of Renal Outcome

Four treatment regimens were used based on the KDIGO guidelines or experience of doctors, including angiotensin receptor blocker (ARB)/angiotensin-converting enzyme inhibitor (ACEI), glucocorticoids, immunosuppressants combined with glucocorticoids, and methylprednisolone + cyclophosphamide pulse therapy. The composite endpoint in our study was defined as a 50% decline in e-GFR, ESRD and/or death. The ESRD was defined as e-GFR <15 mL/min/1.73 m^2^ or the initiation of maintenance renal replacement therapy (haemodialysis, peritoneal dialysis, or renal transplant). The definition of renal insufficiency was e-GFR <60 ml/min/1.73 m^2^. The definition of hypoalbuminemia was that the levels of serum albumin <40 g/L. The definition of hematuria was u-RBC >5 per high power view.

### Statistical Analysis

SPSS package (version 23.0; SPSS Inc., Chicago, IL) and SAS software package version 9.2 (SAS Institute, Cary, NC, USA) were used to perform the statistical analysis. For continuous data, results were presented as mean ± standard deviation or median (interquartile range) based on the covariate distribution whereas categorical variables were expressed in numbers (percentages). Patients were divided into two groups by the optima cut-off value which was calculated by carrying out the ROC curve. Baseline data between groups was assessed using Student's *t*-test, Wilcoxon-test, Chi-square-test or Fisher's exact-test. Kaplan–Meier estimates was used to compute the proportions of endpoint in both matched and unmatched cohorts. Similarly, a Cox regression analysis was adjusted to evaluated the influence of the clinicopathological manifestations on the renal outcome in both cohorts. Subgroup analysis was also carried out, whose heterogeneity was tested by addition of a multiplicative interaction term to the correlative Cox model. ROC was used to verify the prognostic role of serum bilirubin on renal outcomes. All tests were two-sided and P < 0.05 was deemed statistically significant.

## Results

### Selection and the Optimal Cutoff Value

**Figure 1:**
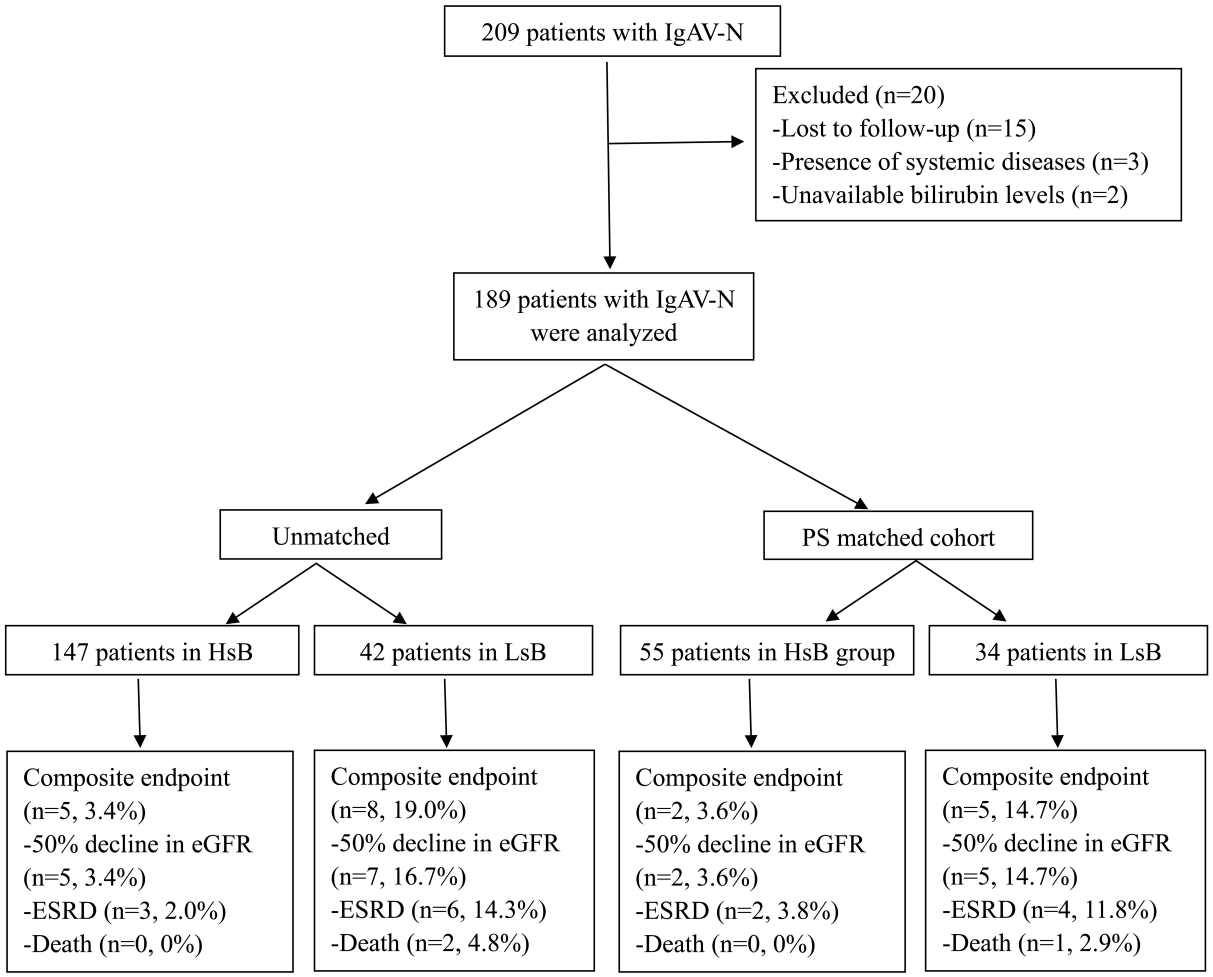
Flow diagram of patient progress and outcomes. IgAV-N, IgA vasculitis with nephritis; PS, propensity score; HsB, high serum bilirubin; LsB, low serum bilirubin; e-GFR, estimate glomerular filtration rate; ESRD, end-stage renal disease.

According to the inclusive and exclusive criteria, 189 patients with IgAV-N (87 men and 102 women) were enrolled in our study (Figure 1). The bilirubin levels of patients in our cohort were ranged from 1.9 to 44.6 umol/L. Only 4 patients had slightly increased bilirubin levels (>28.0 umol/L) while the vast majority of IgAV-N patients were within normal range. The optimal cutoff value of serum total bilirubin calculated by ROC curve was 6.35 umol/L.

Actually, there were many group methods of data handling in statistics, such as ROC and terstiles. In our study, patients were divided into two groups by the optima cut-off value calculated by ROC curve. Many studies have adopted the similar methods (13, 14). Additionally, the terstiles is also a reasonable method to stratify patients. We also performed the analysis based on the interquartile range and the results were similar (Supplementary Information). Notably, the optimal cutoff value of serum total bilirubin calculated by ROC curve was 6.35 umol/L, which was very closed to the first quarter 6.60 umol/L. Due to the lower incidence in adult patients with IgAV-N, only 189 patients were enrolled in our study. It is difficult to make the further statistical analysis according to quartile. Hence, we used the former method.

### Characteristics in Baseline

IgAV-N patients were divided into HsB group (147, 77.8%) and LsB group (42, 22.2%) based on the cutoff value (Table 1). The results suggested that patients in LsB group tended to have higher levels of SBP, proteinuria, glomeruli crescent rates, and serum creatinine and lower levels of serum albumin and hemoglobin. However, other variables were not significantly different (*P* > 0.1). Table 2 demonstrated the treatment options in different groups. No obvious differences were found in both groups except that patients in LsB group tended to have higher rates of pulse therapy (23.8 vs. 12.2%, *P* = 0.063).

**Table 1:** Baseline characteristics of patients categorized according to serum bilirubin levels.

**Characteristics**	**Unmatched cohort**		***P*-values**	**Matched cohort**		***p*-values**
	**HsB group (≥6.35 umol/L) (*n* = 147)**	**LsB group (<6.35 umol/L) (*n* = 42)**		**HsB group (≥6.35 umol/L) (*n* = 55)**	**LsB group (<6.35 umol/L) (*n* = 34)**	
Age	31.3 ± 15	29.0 ± 16.1	0.381	33.1 ± 17.8	26.1 ± 14.2	0.080
Male (%)	66 (44.9)	21 (50.0)	0.559	36 (65.5)	17 (50.0)	0.149
Follow-up (m)	27.4 ± 19.7	26.8 ± 21.8	0.858	27.8 ± 23.2	26.5 ± 22.4	0.860
SBP	121.97 ± 18.42	129.31 ± 18.73	0.024^*^	125.45 ± 17.47	127.09 ± 18.53	0.658
DBP	78.81 ± 11.55	80.95 ± 13.19	0.306	81.60 ± 10.69	80.41 ± 14.22	0.655
HTN (%)	34 (23.1)	12 (28.6)	0.469	15 (27.3)	8 (23.5)	0.695
Proteinuria	1.77 (0.81–2.95)	4.62 (2.92–8.38)	0^**^	3.78 ± 3.44	4.34 ± 3.03	0.442
u-RBC	166.62 ± 432.336	222.07 ± 462.24	0.471	298.38 ± 660.77	116.56 ± 205.16	0.123
ALB	36.89 ± 7.02	29.07 ± 8.71	0^**^	33.71 ± 7.29	30.51 ± 8.70	0.080
SCr	70.11 (58.01–86.25)	69.65 (48.25–117.35)	0.056	79.54 ± 45.64	89.66 ± 55.36	0.352
UA	335.81 ± 101.71	364.22 ± 108.09	0.117	339.04 ± 110.76	356.04 ± 103.42	0.473
e-GFR	110.1 (90.1–128.9)	125.1 (69.7–136.7)	0.219	104.91 ± 37.37	104.46 ± 39.43	0.957
HGB	138.48 ± 18.23	121.40 ± 20.76	0^**^	130.36 ± 17.89	125.65 ± 19.52	0.246
TG	1.74 ± 0.99	1.99 ± 0.98	0.141	1.79 ± 0.84	2.07 ± 1.07	0.178
CHOL	5.15 ± 1.66	5.58 ± 1.94	0.149	5.66 ± 1.78	5.66 ± 1.90	0.993
T-BIL	11.3 (8.5–14.6)	4.8 (3.9–5.7)	0^**^	10.5 (8.4–14.2)	4.5 (3.9–5.7)	0^**^
D-BIL	3.1 (2.45–4.1)	1.4 (1.1–1.6)	0^**^	3.1 (2.1–4.1)	1.4 (1.0–1.6)	0^**^
I-BIL	7.9 (6.4–10.8)	3.2 (2.7–4.1)	0^**^	7.5 (6.1–10.2)	3.1 (2.7–4.1)	0^**^
M0/M1 (%)	21/126 (14.3/86.7)	7/35 (16.7/83.3)	0.702	4/51 (7.3/92.7)	6/28 (17.6/82.4)	0.132
E0/E1 (%)	122/25 (83/17)	35/7 (83.3/16.7)	0.959	45/10 (81.8/18.2)	30/4 (88.2/11.8)	0.419
S0/S1 (%)	96/51 (65.3/34.7)	24/18 (57.1/42.9)	0.333	39/16 (70.9/29.1)	20/14 (58.8/41.2)	0.284
C0/C1 (%)	96/51 (65.3/34.7)	20/22 (46.7/52.4)	0.038^*^	27/28 (49.1/50.9)	17/17 (50.0/50.0)	0.934
T0/T1 (%)	86/61 (58.5/41.5)	26/16 (61.9/38.1)	0.692	29/26 (52.7/47.3)	20/14 (58.8/41.2)	0.574

*SBP, systolic blood pressure; DBP, diastolic blood pressure; HTN, hypertension; u-RBC, uric red blood cell; ALB, albumin; SCr, serum creatinine; UA, uric acid; e-GFR, estimated glomerular filtration rate; HGB, hemoglobin; TG, triglyceride; CHOL, cholesterol; T-BIL, total bilirubin; D-BIL, direct bilirubin; I-BIL, indirect bilirubin; M, mesangial proliferation; E, endocapillary proliferation; S, segmental glomerulosclerosis; C, crescents; T, tubular atrophy/interstitial fibrosis*.

* *Stands for p < 0.05*.

** *Stands for p ≤ 0.01*.

**Table 2:** Principal treatments of patients with HSPN.

**Characteristics**	**Unmatched cohort**		***P-*values**	**Matched cohort**		***P*-values**
	**HsB group (≥6.35 umol/L) (*n* = 147)**	**LsB group (<6.35 umol/L) (*n* = 42)**		**HsB group (≥6.35 umol/L) (*n* = 55)**	**LsB group (<6.35 umol/L) (*n* = 34)**	
ACEI/ARB (%)	16 (10.9)	1 (2.4)	0.126	3 (5.4)	1 (2.9)	1.000
Glucocorticoids (%)	61 (41.5)	14 (33.3)	0.340	22 (40.0)	14 (41.2)	0.913
Glucocorticoids + Immunosuppressants (%)	52 (36.4)	17 (40.5)	0.545	20 (36.4)	10 (29.4)	0.5000
Pulse therapy with methylprednisolone and cyclophosphamide (%)	18 (12.2)	10 (23.8)	0.063	10 (18.2)	9 (26.5)	0.354

*ACEI, angiotensin-converting enzyme inhibitor; ARB, angiotensin II receptor blocker*.

PS matches was performed to balance the baseline covariates, which might potentially influence the outcomes. Thirty-four patients (17 men and 17 women) were assigned to the LsB group while 55 patients (36 men and 19 women) were categorized into the HsB group. PS was derived from a multi-logit regression model with parameters including SBP, excretion levels of 24-h urinary protein, serum albumin, creatine, hemoglobin, presentation of crescents and so on, then there were no differences in clinicopathological manifestations and treatments except serum bilirubin levels between groups (Tables 1, 2).

### Outcomes of Renal Survival

**Figure 2:**
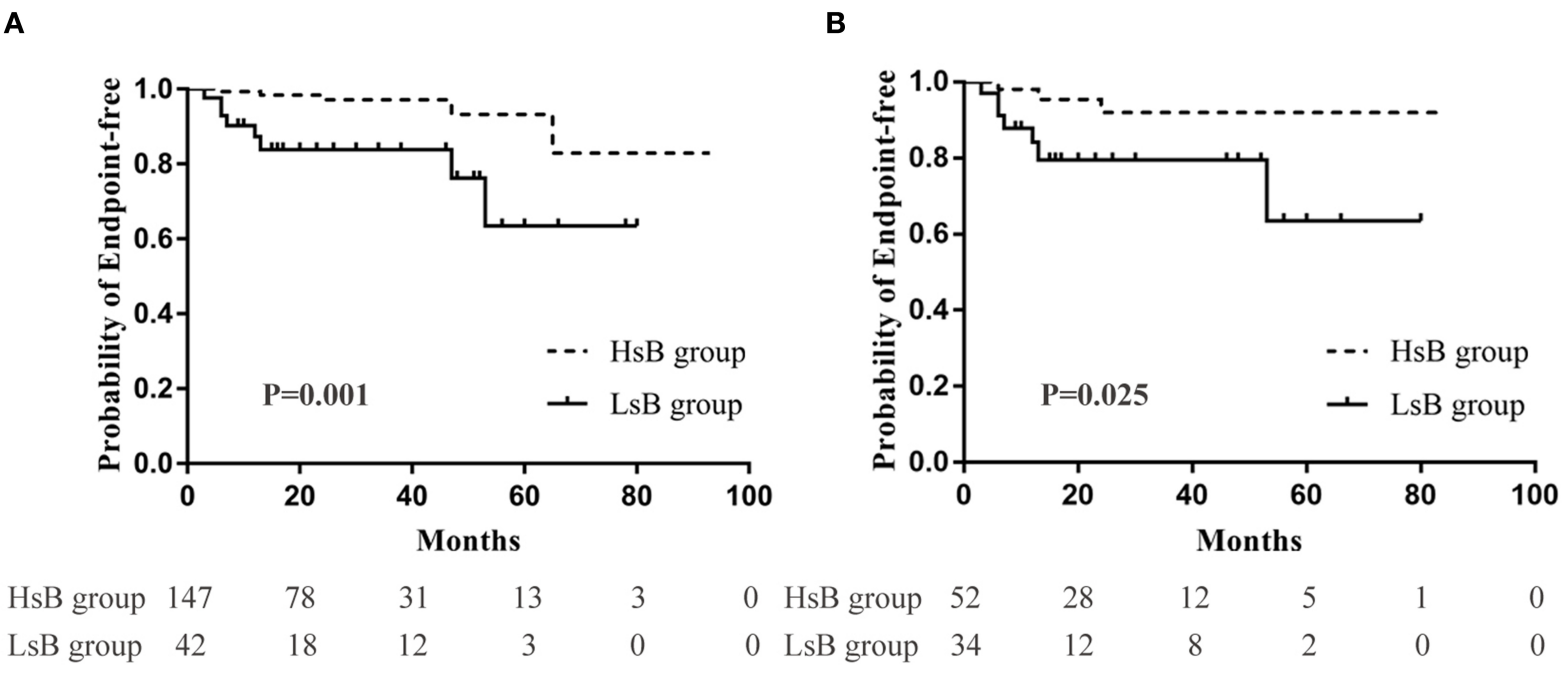
Renal outcomes between high serum bilirubin (HsB) group and low serum bilirubin (LsB) group. **(A)** Kaplan-Meier curve for outcomes in the unmatched and **(B)** the matched cohort.

In the unmatched cohort, Kaplan-Meier analysis demonstrated that 3.4% (5 out of 147) and 19.0% (8 out of 42) patients reached the composite endpoints in the HsB group and the LsB group, respectively (Figure 2A). This result suggested that patients with lower levels of serum bilirubin seemed to have a worse outcome (*P* = 0.001). Similar result was observed in the matched cohort, 3.6% (2 out of 55) and 14.7% (5 out of 34) reached the composite endpoints (*P* = 0.050, Figure 2B).

### Prognostic Role of Serum Bilirubin

Multivariate Cox analysis adjusted for clinicopathological confounding factors such as SBP, diastolic blood pressure (DBP), serum albumin, e-GFR, cholesterol, proteinuria, u-RBC, anemia and pathological changes (MESTC), strongly illustrated that serum bilirubin was an independent protective factor of prognosis in both unmatched cohort (HR = 0.17, 95% CI, 0.03–0.99; *P* < 0.05) and matched cohort (HR = 0.05, 95% CI, 0.00–1.03; *P* = 0.050) (Table 3).

**Table 3:** Risk factors for renal outcomes by a cox-regression analysis categorized according to serum bilirubin levels.

	**Unmatched cohort**		**Matched cohort**	
	**HR (95%CI)**	***p***	**HR (95%CI)**	***p***
BIL (high/low)	0.17 (0.03–0.99)	0.049^*^	0.05 (0.00–1.03)	0.050^*^
HTN	1.58 (0.27–9.37)	0.613	20.25 (0.23–1758.10)	0.187
Proteinuria (g/d)	1.20 (0.91–1.58)	0.197	1.60 (0.83–3.10)	0.159
e-GFR (ml/min/1.73 m^2^)	1.00 (0.98–1.02)	0.924	1.00 (0.98–1.03)	0.831
u-RBC (/HP)	1.00 (0.99–1.00)	0.292	1.00 (0.99–1.00)	0.655
ALB (g/L)	0.95 (0.84–1.08)	0.468	0.92 (0.70–1.20)	0.521
CHOL (mmol/L)	1.34 (0.92–1.97)	0.131	1.10 (0.74–1.63)	0.641
Anemia	4.83 (0.81–28.97)	0.085	2.09 (0.06–73.04)	0.685
M (M0/M1)	0.24 (0.01–4.00)	0.318	0.05 (0.00–7.07)	0.237
E (E0/E1)	0.96 (0.12–8.05)	0.960	2.1E7 (0.00–5.8E7)	0.981
S (S0/S1)	0.47 (0.11–2.05)	0.132	0.20 (0.02–1.99)	0.171
C (C0/C1-2)	5.44 (0.81–36.65)	0.082	0.65 (0.02–27.34)	0.823
T (T0/T0-1)	0.01 (0.00–0.10)	0.000^**^	0.00 (0.00–0.29)	0.013^*^

*BIL, the high serum bilirubin group/the low serum bilirubin group; HTN, hypertension; e-GFR, estimated glomerular filtration rate; u-RBC, the count of uric red blood cell; ALB, albumin; CHOL, cholesterol; M, mesangial proliferation; E, endocapillary proliferation; S, segmental glomerulosclerosis; C, crescents; T, tubular atrophy/interstitial fibrosis*.

* *Stands for p < 0.05*.

** *Stands for p ≤ 0.01*.

**Figure 3:**
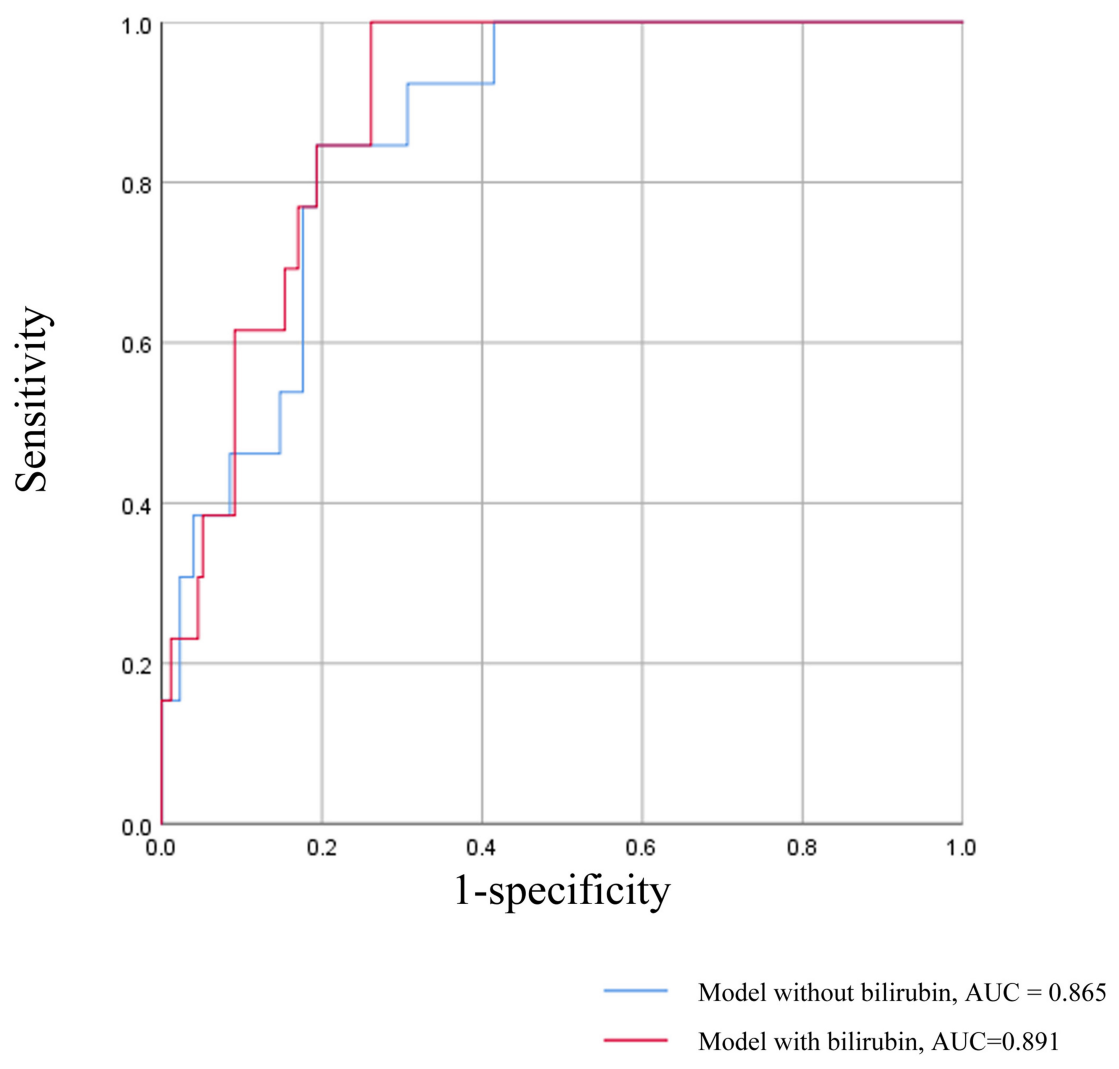
ROC curves for the survival model in prediction of the poor renal prognosis. The survival model comprising the variables of serum bilirubin, age at biopsy, blood pressure, proteinuria, albumin, e-GFR, MEST score, and treatment.

A survival model comprising the variables of serum bilirubin, age at biopsy, blood pressure, proteinuria, albumin, e-GFR, MEST score, and treatment was established to illustrate the predictive power of serum bilirubin, which was measured by ROC curves (Figure 3). The AUC value of the survival model was 0.891. But when serum bilirubin was removed, the AUC value dropped to 0.865. Accordingly, the discrimination of poor prognosis could be improved by adding the clinical indicators of serum bilirubin.

**Figure 4:**
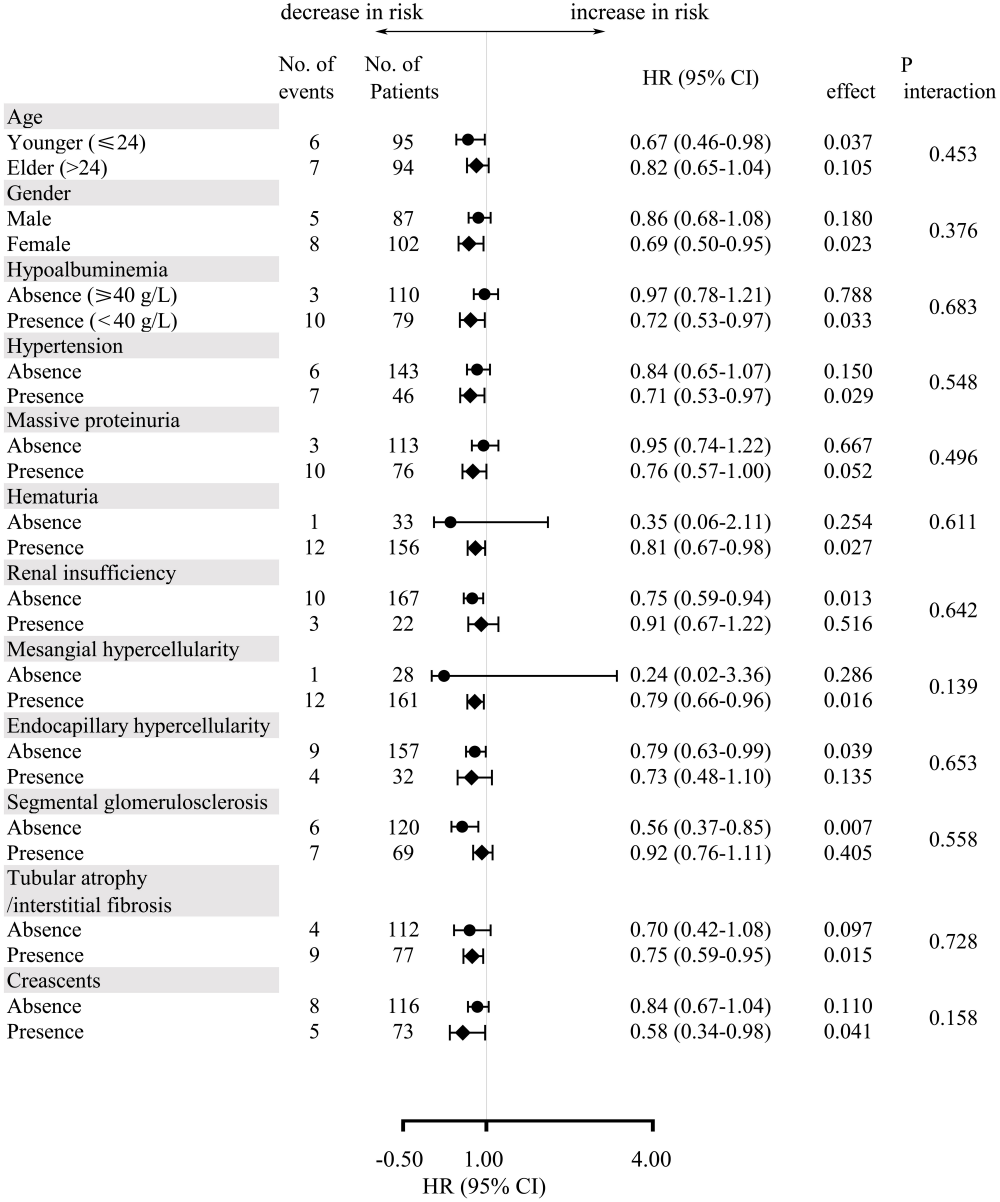
Multivariate-adjusted hazard ratios and 95% confidence intervals for the development of composite endpoints for every 1 umol/L increase in serum bilirubin according to subgroups of baseline characteristics.

Subgroup analysis stratified by potential confounders in unmatched cohort was carried out to verify the consistency of the correlation between serum bilirubin and the renal outcomes (Figure 4). The multivariate-adjusted hazard ratios demonstrated that there was a trend that every 1 umol/L increase would protect IgAV-N patients from the composite endpoints. Moreover, baseline characteristics including age, gender, hypoalbuminemia, hypertension, massive proteinuria, hematuria, renal insufficiency, M, E, S, T, and C, had no multiplicative interaction with serum bilirubin (*P* > 0.10). Similar results had also been found in the matched cohort.

### Renal Survival Adjusted for Anemia and T Injury

**Figure 5:**
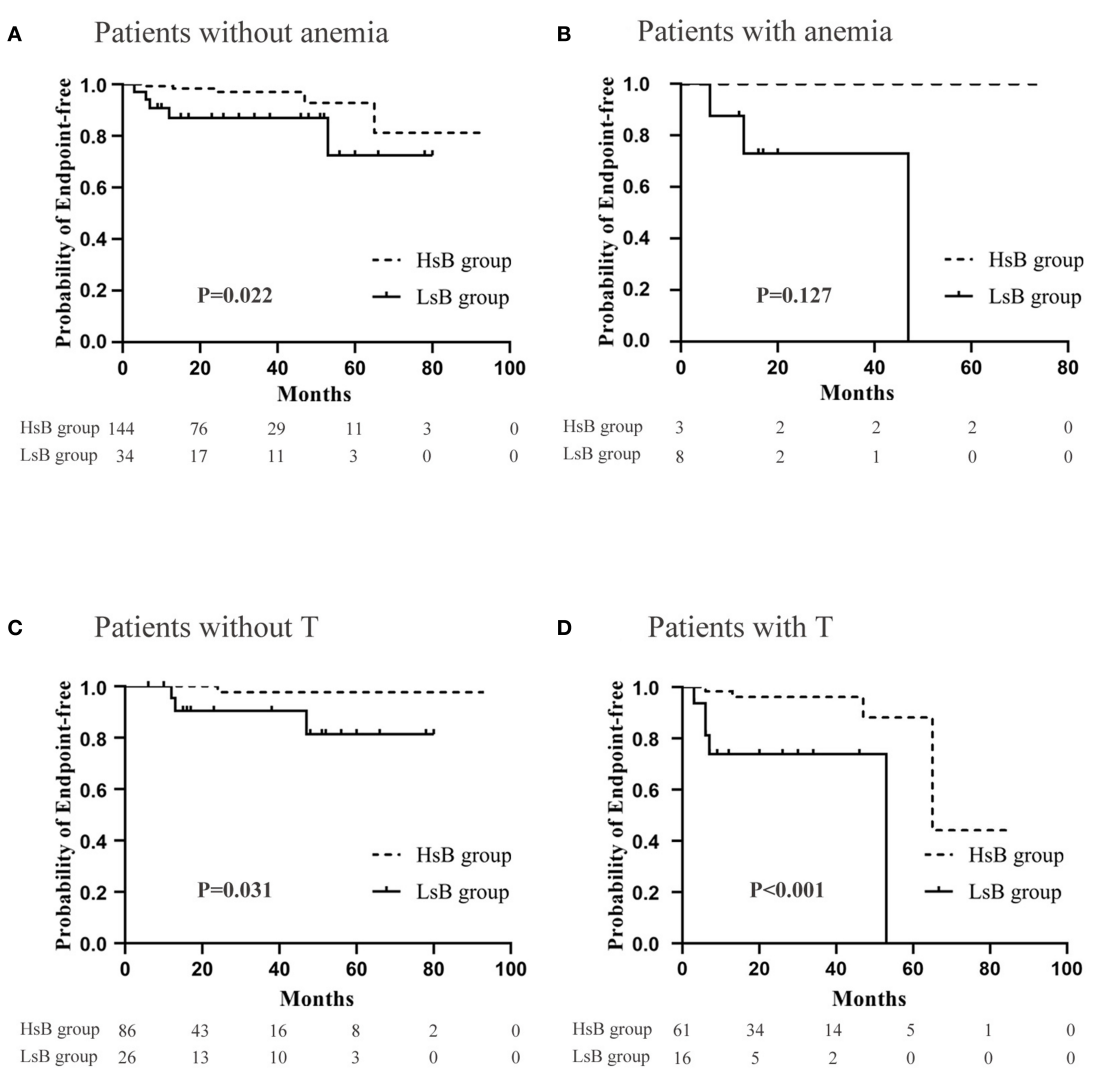
Renal outcomes between high serum bilirubin (HsB) group and low serum bilirubin (LsB) group adjusted for anemia and tubular atrophy/interstitial fibrosis. **(A)** Patients without anemia. **(B)** Patients with anemia. **(C)** Patients without T. **(D)** Patients with T.

It has been well-acknowledged that anemia and tubular atrophy/interstitial fibrosis might result in an adverse prognosis. And the predictive role of bilirubin, a degradation product of heme, might be also influenced by anemia and tubular atrophy/interstitial fibrosis. Therefore, subgroup analysis was carried out in order to eliminate their potential impacts. It was easily found that patients in HsB group had a significantly better prognosis (Figure 5). Notably, the difference of prognosis between HsB group and LsB group in patients with anemia was not statistically significant (*P* = 0.127). These resulted from the small sample size but we could already see the trend that patients with lower level of serum bilirubin had more unfavorable outcomes. Hence, it was quite reasonable to arrive at the conclusion that serum bilirubin could serve as an independent predictor in IgAV-N regardless of anemia and tubular atrophy/interstitial fibrosis.

## Discussion

IgAV-N is a common vasculitis in children, whose morbidity is about 10/100,000 every year. There are few high-quality researches of adolescent and adult IgAV-N due to a lower incidence in them. So, the present study was conducted to find out an effective prognostic marker to help clinicians have a better understanding of adolescent-onset and adult-onset IgAV-N. To our knowledge, this study is the first one to demonstrate that serum bilirubin levels could serve as an independent protective factor of IgAV-N progression. The bilirubin levels of the vast majority of IgAV-N patients in our cohort were within normal range. In this study, we found that patients with lower levels of serum bilirubin often presented with more severe clinical manifestations such as hypoproteinemia, hypertension, large amount of proteinuria, and renal dysfunction. Conversely, patients with higher serum bilirubin level tended to have a better prognosis, with only 3.4% patients reaching the composite endpoints. Moreover, multivariate Cox regression analysis also proved a strong protective effect of serum bilirubin on renal survival (HR = 0.198, 95% CI, 0.041–0.955; *P* < 0.05), adjusted for confounding factors including SBP, DBP, serum albumin, e-GFR, cholesterol, proteinuria levels, u-RBC, and renal pathological changes. Consistent with our findings, similar relationship between bilirubin and e-GFR were found previously in diabetes patients (15, 16). Considering that different baseline data might contribute to discrepancies, PS matches were applied to make the indicators more comparable. After that, similarly, 20.6% patients in the LsB group had remarkable deterioration of renal function while only 5.8% in the HsB group, with confirmed the renal protection effect of higher serum bilirubin levels.

Several literatures have reported that higher serum bilirubin level could prevent adverse renal outcomes in CKD (5, 17, 18). A meta-analysis also illustrated that the risk of progressing to CKD might get decreased due to the elevated bilirubin levels (6). In addition, Tanaka et al. found that serum bilirubin levels were negatively correlated to pathological findings based on Oxford classification of IgAN (4). In our study, the proportion of presentation of glomeruli cellular/fibrocelluar crescents in patients of LsB group was distinctly higher (52.4 vs. 34.7%, *P* = 0.038), which might lead to the poor renal outcome.

Considering the multiple function of bilirubin, it is hypothesized that bilirubin might affect the renal prognosis of IgAV-N through three main effects, which are antioxidant effects, anti-inflammatory effects, and endothelial protection. Firstly, it has been proved that increased oxidative stress contributes significantly to the development of CKD and reactive oxygen species (ROS) is permeated in the whole process of tissue damage in IgAV-N (19, 20). Bilirubin is a potent endogenous antioxidant and cellular protectant, which is capable of scavenging free radical production and prevent oxidation reactions observed in glomerulonephritis such as IgAN (21). Secondly, serum bilirubin is shown to possess potent complement inhibitory and anti-inflammatory effects. Complement activation play a crucial part in the pathogenesis of IgAV-N. Higher serum bilirubin could inhibit the complement activation and inflammatory cells infiltration. The anti-inflammation effect of bilirubin can be explained by the decreased chemoattractant and cytokine levels, the inhibition of complement and other potential mechanisms, which substantially diminish the infiltration of inflammatory cells like neutrophils (22). Thirdly, it is known that IgAV-N is a common vasculitis with prominent vascular damage. Further evidences have proved a positive correlation between ESRD and endothelial dysfunction, which can potentially give rise to atherosclerosis, vascular stenosis and hypertension (23, 24). It was reported that moderate higher level of bilirubin has a great advantage on endothelial protection and antihypertensive effects (25). Therefore, IgAV-N patients might benefit from the relatively elevated bilirubin concentration.

In theory, adding bilirubin appropriately has renoprotective effects on IgAV-N patients, which might be a new therapeutic method. Phycocyanobilin, an organic matter obtained from phycocyanin, could combine with biliverdin reductase to give rise to an analog of bilirubin. An animal experiment carried out by Zheng et al. suggested that oral administration of phycocyanobilin could avoid diabetic mice suffering from diabetic nephropathy (7). Unfortunately, the evidence that IgAV-N patients could get benefit from it is still lacking. Consequently, further studies are required to clarify whether low serum bilirubin could serve as a future therapeutic target.

There are several limitations in our study. Firstly, individuals were recruited in a single medical center with a relatively small sample size and all the subjects were Han Chinese. Hence, it might not be perfectly safe to apply our findings to other ethnic groups. Secondly, our study was based on clinical observation, intervene experiments may be needed to further verify the hypothesis. Finally, potential confounders adjusted in this study were limited. Other unmeasured confounders like insulin resistance, treatment regimens and smoking status could not be eliminated in the present study.

## Conclusion

To sum up, elevated bilirubin levels are correlated to the favorable renal outcomes. Further studies are required to clarify whether IgAV-N patients could get benefit from administration of bilirubin.

## Data Availability Statement

The datasets presented in this study can be found in online repositories. The names of the repository/repositories and accession number(s) can be found in the article/supplementary material.

## Ethics Statement

The studies involving human participants were reviewed and approved by the Ethics Committee of the West China Hospital of Sichuan University. Written informed consent to participate in this study was provided by the participants' legal guardian/next of kin.

## Author Contributions

GP and JT: conception and design of the study, collection and analysis of data, drafting of the article, critical revision for important intellectual content, and final approval of the version to be published. YT, YX, and TH: collecting the follow-up data, analysis and interpretation of statistics, critical revision for important intellectual content, and final approval of the version to be published. LT, ZZ, and PT: analysis and interpretation of data, critical revision for important intellectual content and final approval of the version to be published. WQ and YT: the presenter of this project, who put forward the concept and designed the study, revised the important intellectual content critically and agrees to publish the final version. All authors contributed to the article and approved the submitted version.

## Conflict of Interest

The authors declare that the research was conducted in the absence of any commercial or financial relationships that could be construed as a potential conflict of interest.
