# Supplementary material for: Serum Bilirubin Is Correlated With the Progression of IgA Vasculitis With Nephritis
Source: Front Med (Lausanne). 2021 Jun 8;8:596151. doi: 10.3389/fmed.2021.596151 (PMC8217633; doi:10.3389/fmed.2021.596151)

Supplementary Information. Terstiles of total bilirubin concentration and renal outcomes based on the interquartile range.

| 5% | 10% | 25% | 50% | 75% | 90% | 95% |
| --- | --- | --- | --- | --- | --- | --- |
| 3.85 | 4.50 | 6.60 | 9.50 | 12.55 | 17.20 | 21.70 |


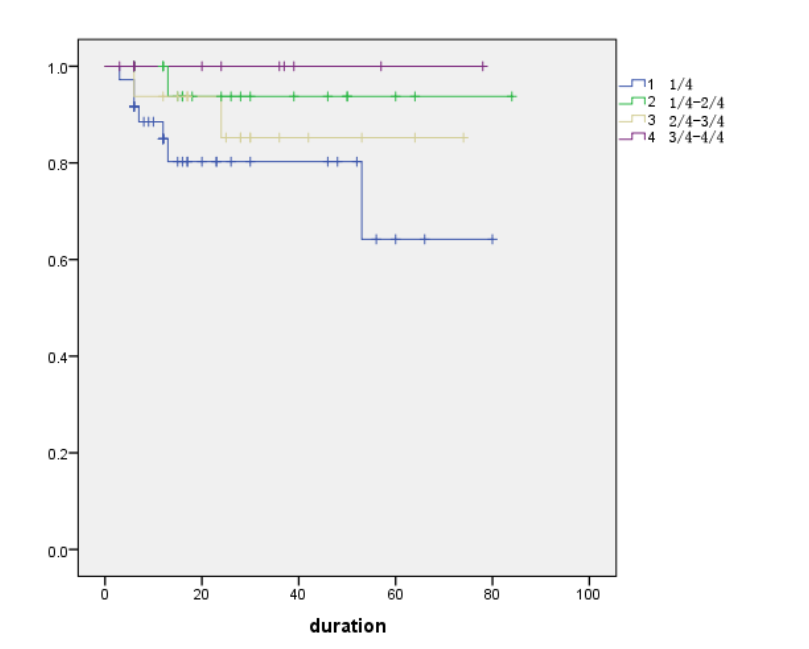

Supplement: Supplementary file 1 [file Data_Sheet_1.docx]
